# Updated *Campylobacter jejuni* Capsule PCR Multiplex Typing System and Its Application to Clinical Isolates from South and Southeast Asia

**DOI:** 10.1371/journal.pone.0144349

**Published:** 2015-12-02

**Authors:** Frédéric Poly, Oralak Serichantalergs, Janelle Kuroiwa, Piyarat Pootong, Carl Mason, Patricia Guerry, Craig T. Parker

**Affiliations:** 1 Enteric Diseases Department, Naval Medical Research Center, Silver Spring, MD, United States of America; 2 Armed Forces Research Institute of Medical Sciences, Bangkok, Thailand; 3 United States Department of Agriculture, Agricultural Research Service, Albany, CA, United States of America; University of Helsinki, FINLAND

## Abstract

*Campylobacter jejuni* produces a polysaccharide capsule that is the major determinant of the Penner serotyping scheme. This passive slide agglutination typing system was developed in the early 1980’s and was recognized for over two decades as the gold standard for *C*. *jejuni* typing. A preliminary multiplex PCR technique covering 17 serotypes was previously developed in order to replace this classic serotyping scheme. Here we report the completion of the multiplex PCR technology that is able to identify all the 47 Penner serotypes types known for *C*. *jejuni*. The number of capsule types represented within the 47 serotypes is 35. We have applied this method to a collection of 996 clinical isolates from Thailand, Cambodia and Nepal and were able to successfully determine capsule types of 98% of these.

## Introduction

*Campylobacter jejuni* is among the leading causes of bacterial diarrheal disease worldwide. In the U.S., *Campylobacter* is currently the second cause of foodborne bacterial disease behind Salmonella with an estimated incidence of 14.22 cases per 100,000 million annually, affecting all age classes [1]. In developing countries, the incidence is higher and predominantly affects children less than two years old [2, 3]. It is estimated that 5.5 to 18% of children under the age of 5 years develop diarrhea caused by this pathogen [4]. In addition, *C*. *jejuni* is associated with several sequelae, including irritable bowel syndrome (IBS) [5, 6], reactive arthritis [7], and Guillain-Barré syndrome [8]. Moreover, recent studies suggest the association of repeated *C*. *jejuni* infections with malnutrition and stunting [9].

**Fig 1:**
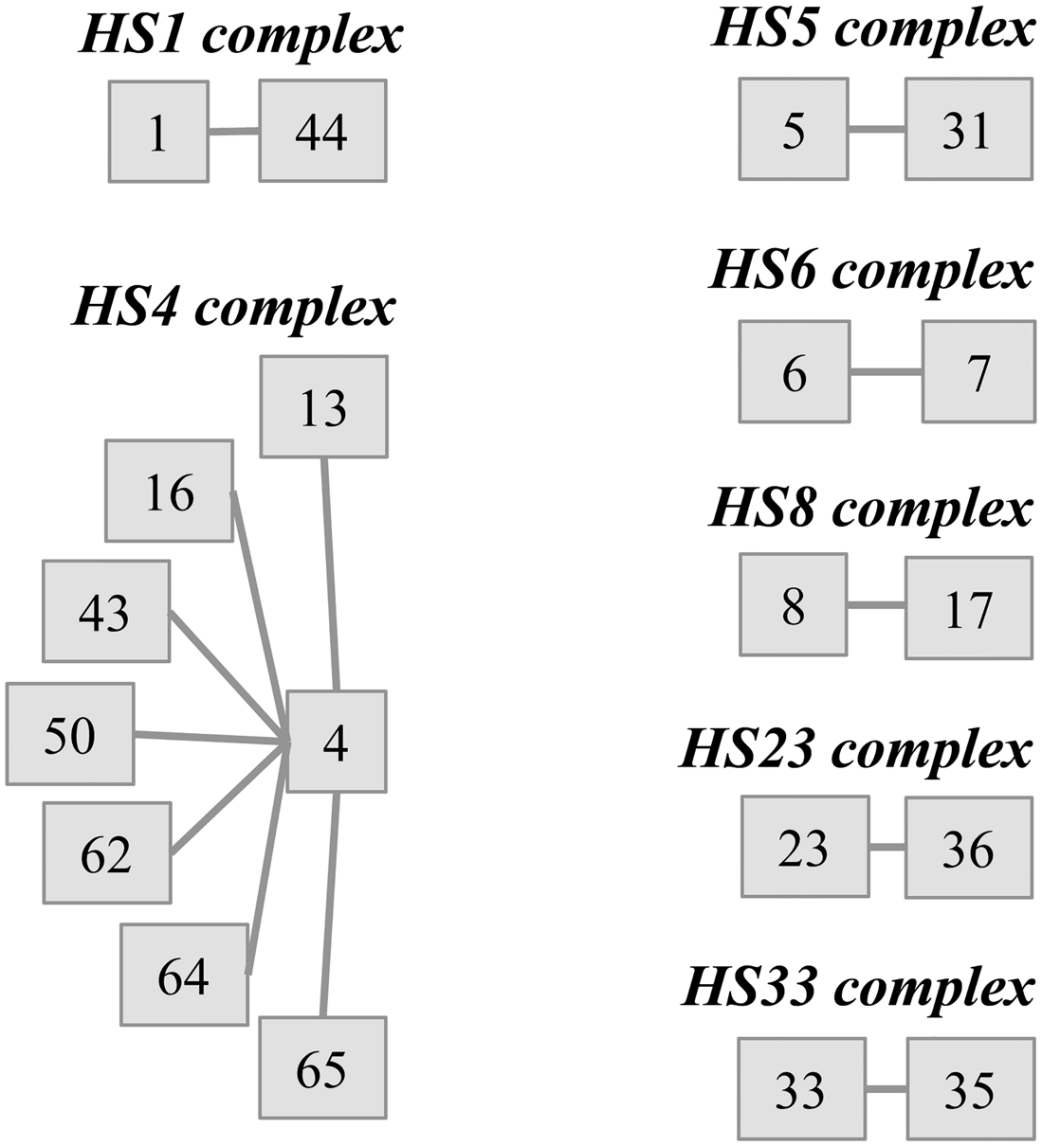
This cartoon summarizes the Penner types association into complexes commonly found in literature. The variable CPS biosynthesis loci of type strains belonging to the same complex present a high degree of homology [31, 32].

A decade of research utilizing whole genome sequencing and Comparative Genomic Hybridization (CGH) using whole genome microarray analyses has revealed extensive genetic variability among *C*. *jejuni* strains [10–19]. The most variable genetic loci are those involved in the synthesis and modification of bacterial surface carbohydrate structures including the genes involved in flagellar O-linked glycosylation, genes involved in biosynthesis of lipooligosachharide (LOS), and the polysaccharide capsule (CPS) [10], all of which contribute to virulence in a variety of ways. Non-encapsulated mutants are defective in colonization of chickens and mice [20, 21], and show reduced virulence in infant ferret [22]. Moreover, the CPS is required for resistance to complement-mediated killing [21–24]. CPS is also the primary determinant of the Penner or heat-stable serotyping scheme, of which there are 47 *C*. *jejuni* serotypes [25, 26], some of which fall into complexes of related serotypes (Fig 1) [25, 27, 28]. *C*. *jejuni* CPSs are exported via a highly conserved ABC-transporter mechanism similar to class 2 and class 3 capsules of *E*. *coli* [29]. The variable genes that encode the enzymes responsible for synthesis of the serotype-specific CPS are located between two blocks of ABC transporter genes (*kpsMTEDF* at one end of the locus, and *kpsCS* at the other end. To date the sequence of 18 of these variable CPS loci have been published [10, 15, 19, 30–32], and the remainder are in preparation (C. T Parker, unpublished; F. Poly, unpublished). We are currently evaluating a polysaccharide CPS conjugate approach against *C*. *jejuni* -mediated disease [29, 33, 34]. A final vaccine formulation, like most other polysaccharide conjugate vaccines would be multivalent [35]. The criteria for inclusion of specific CPS types into a multivalent formulation would be based on both incidence of specific CPS types and any association of specific CPS types with severity of illness. However, a recent systematic review of published studies on Penner serotyping of strains isolated from 1978–2002 demonstrated a paucity of data from developing countries, where a vaccine is the most needed [27]. Additional information on CPS types present in endemic areas is clearly needed to facilitate development of a multivalent conjugate vaccine approach. Unfortunately, the complexity and costs of Penner serotyping have limited its use in recent years. To circumvent this problem, a partial multiplex PCR methodology was successfully introduced in order to gain more information on *C*. *jejuni* CPS distribution worldwide [32]. The initial methodology was able to distinguished 17 of the more frequently isolated CPS types. In this report we present an updated version of the multiplex PCR that is now able to identify all known CPS *C*. *jejuni* types. These data indicate that the 47 serotypes can be collapsed into 35 CPS types (Fig 1). Here, we also describe application of this method to a collection of 996 *C*. *jejuni* clinical isolates collected from South and Southeast Asia from 1998 to 2010.

## Materials and Methods

### DNA purification

Frozen stocks of *C*. *jejuni* were subcultured on Brucella agar plates with 5% sheep blood (BAP) and incubated at 37°C under microaerobic conditions (5% O_2_, 10% CO_2_, 85% N_2_). Genomic DNA extractions of *C*. *jejuni* isolates were performed using DNeasy tissue extraction kits (Qiagen, USA) following the manufacturer’s instructions. Genomic DNAs were routinely stored at -20°C.

### Whole genome sequencing and CPS loci annotation

**Fig 2:**
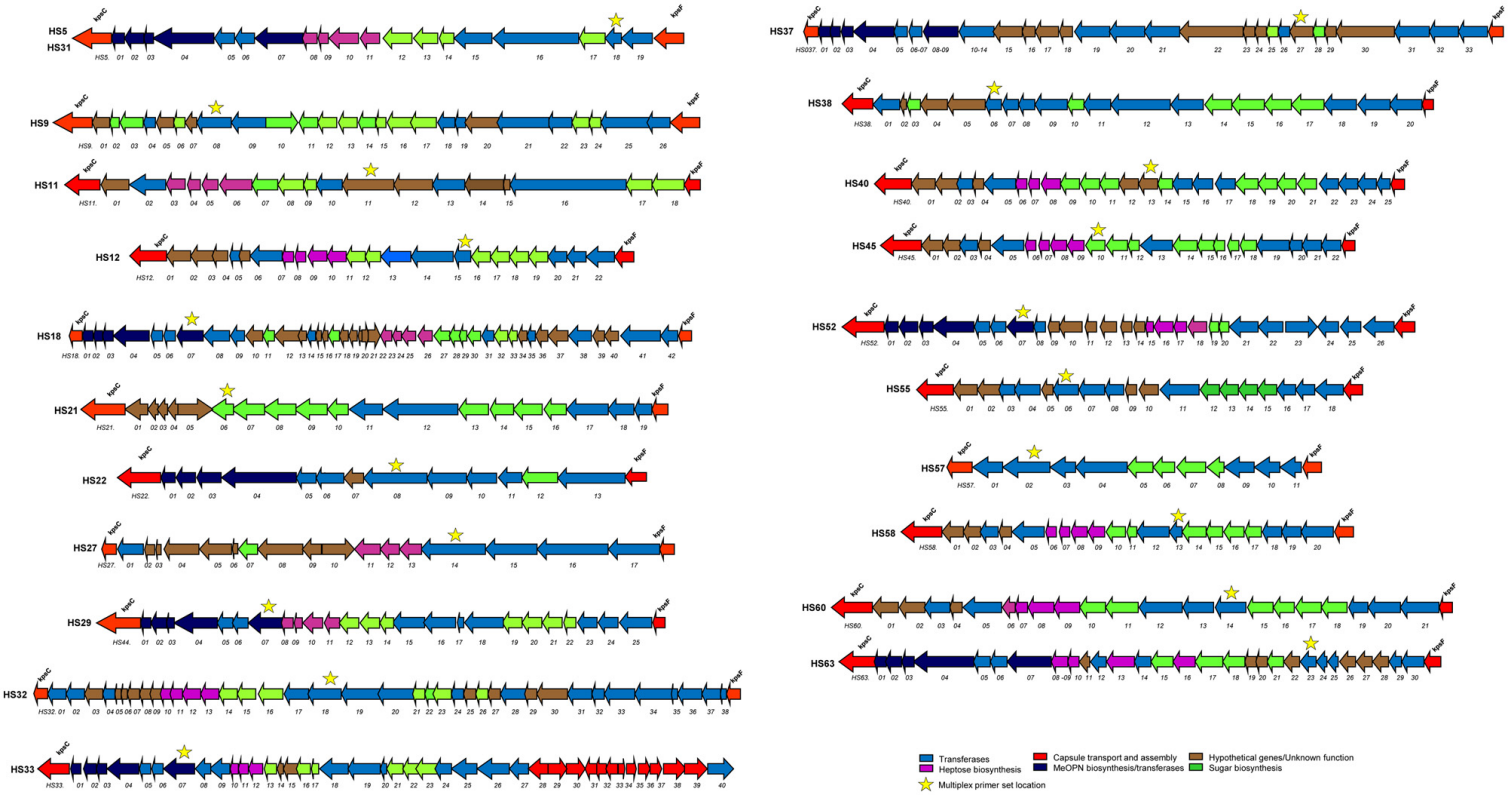
Illustration of the *C*. *jejui* CPS loci described in this study. Putative gene functions were assigned via homology to a protein database by BLAST analyses.

CPS loci sequences were extracted from the USDA-ARS CRIS 5325-42000-047 project aimed at the whole genome sequencing of 45 *C*. *jejuni* Penner type strains. Strains are shown in Table 1. Whole-genome sequencing of the 45 *C*. *jejuni* Penner type strains to a depth of ~20x was performed using shotgun and paired-end (8 to 12 kb) libraries and was generated on a Roche 454 FLX+ sequencing system with Titanium chemistry. The Roche Newbler assembler (version 2.3) was used to assemble reads into contigs. Genome closing utilized a combination of steps. The contigs were aligned to other *C*. *jejuni* genomes. Scaffold gaps were filled by a combination of referenced assemblies of approximately one million Illumina MiSeq reads/strain to the Newbler contigs using Geneious software (Biomatters, New Zealand) and the identification of repeated contigs using the Perlscript contig_extender2. Certain gaps were validated using PCR amplification and Sanger sequencing. All base calls were validated using the Illumina MiSeq reads, which provided an additional 100× coverage. Shotgun library preparations and sequence procedures were performed according to established procedures and manufacturer’s instructions. Annotation of the variable region of CPS biosynthesis, between *kpsC* and *kpsF* (Fig 2), was made using Artemis software (Sanger institute).

**Table 1:** Bacterial strains used for analysis and validation of the capsule multiplex PCR.

Strain	Penner type	Reference
ATCC 43429	HS1	[36]
NCTC 11168	HS2	[10]
ATCC 43431	HS3	[36]
ATCC 43432	HS4	[36]
GC8486	HS4/13/64	[14]
ATCC 43433	HS5	[36]
81116	HS6	[37]
ATCC 43435	HS7	[36]
ATCC 43436	HS8	[36]
ATCC 43437	HS9	[36]
ATCC 43438	HS10	[36]
RM3415	HS11	[38]
RM3204	HS12	[38]
ATCC 43441	HS13	[36]
ATCC 43442	HS15	[36]
RM3417	HS16	[38]
ATCC 43444	HS17	[36]
RM3419	HS18	[38]
ATCC 43446	HS19	[36]
ATCC 43447	HS21	[36]
ATCC 43448	HS22	[36]
81–176	HS23/36	[39]
RM3423	HS27	[38]
RM3424	HS29	[38]
ATCC 43452	HS31	[36]
RM3425	HS32	[38]
ATCC 43454	HS33	[36]
RM3426	HS35	[38]
RM3428	HS37	[38]
RM3429	HS38	[38]
ATCC 43459	HS40	[36]
ATCC 43460	HS41	[36]
ATCC 43461	HS42	[36]
ATCC 43463	HS44	[36]
RM3432	HS45	[38]
ATCC 43465	HS50	[36]
RM3434	HS52	[38]
RM1221	HS53	[40]
ATCC 43469	HS55	[36]
RM3436	HS57	[38]
ATCC 43470	HS58	[36]
RM3438	HS60	[38]
RM3439	HS62	[38]
RM3440	HS63	[38]
ATCC 49302	HS64	[36]
RM3442	HS65	[38]

### PCR primer design

Selection of CPS regions for primer design was performed as previously described [31, 32]. Briefly, specific CPS sequences (variable capsule region between *kpsC* and *kpsF*) for a particular serotype were isolated by performing a local stand alone BLAST using a database encompassing the nucleotides sequences of all 47 available *C*. *jejuni* capsule loci (C. T. Parker, unpublished). The selected nucleotide regions were used for multiplex primer design. The sites where the primers were designed are displayed in Fig 2. Multiplex primers were designed via the online software Primer3 [41] using the following parameters: length between 18 and 30 residues, 20 to 50% GC, T_m_ ranging from 57 to 63°C. Primer sets to be included in the original or new multiplex mixes were designed in order to amplify a PCR of least 20 base pairs smaller/larger than the other amplicons of the same mix. Following design, primers were compared to *C*. *jejuni* genomes via NCBI BLAST software to exclude potential amplification outside the CPS locus.

### Multiplex PCR parameters

PCRs were performed using 0.4 μM of each primers of their respective mixes in 25-μl reactions containing 1–10 ng DNA template, 1× PCR Gold Buffer, 2.0 mM MgCl_2_, 0.3 mM each dNTP and 1.25 U of AmpliTaq Gold DNA Polymerase (Life Technologies, USA) using Dyad thermal cycler (Bio-Rad, USA). DNA amplification was performed using an initial denaturation step at 94°C for 5 min; followed by 30 cycles of amplification (denaturation at 94°C for 1 min, annealing at 52°C for 1 min, and extension at 72°C for 1 min and ending with a final extension at 72°C for 10 min. At the end of the reaction, the PCR amplicons were analyzed by gel electrophoresis on 10-cm-long 2% agarose gels in 0.5× TBE (Tris-borate-EDTA) buffer at 175 V for 75 min. The serotypes were determined by the size of the PCR amplicons by comparison with a 100-bp molecular size standard (New England BioLabs, USA).

### Validation of PCR primers and multiplex mixes

Primer sets were validated individually on their respective DNA type, and, if an amplicon of the predicted size was observed, the primer set was included in its multiplex mix. The newly generated multiplex mix was then used in a PCR reaction on a collection of 47 *C*. *jejuni* DNA (Table 1). The newly designed primer pair was selected and incorporated in multiplex mixes only if it yielded the right size amplicon during PCR performed on its target or related DNA CPS type (i.e. a strain that was part of the same complex) and if no false amplification was observed on the remaining CPS types tested. The newly formulated multiplex mix was finally tested against the DNA of 47 CPS types individually and deemed worthy only if the expected right size amplicons were obtained and no false positives were observed. There are four exceptions to this rule, as discussed below.

### Clinical *C*. *jejuni* isolates

A total of 996 archived *C*. *jejuni* isolates were included in this study. These isolates were from twelve studies on etiology of diarrhea among travelers, military personnel and indigenous population from Southeast Asia during 1998–2010 as shown in Table 2. All stool samples were routinely cultured for enteric bacteria pathogens at the Armed Forces Research Institute of Medical Sciences (AFRIMS). The Cobra Gold exercises in 1998–2003 were approved by ethical review committees from Walter Reed Army Institute of Research (WRAIR) IRB. The travelers’ diarrhea and diarrhea surveillance studies in Thailand, Cambodia and Nepal were approved by ethical review committees from WRAIR IRB as well as host nation IRB (Thailand, Cambodia and Nepal). The studies involve using de-identify archived frozen *C*. *jejuni* isolated from stool samples with appropriate consent for samples donation in the future use and currently stored at the Department of Enteric Diseases, AFRIMS without any identifiable information. The studies were closed. Specimens are labeled by subject numbers and date of collection without any personal identifiers. A link to subject name, number, and personal identifier was destroyed. Data to be included in the analysis portion of this study will be demographic (age and gender), clinical (associated symptoms), and laboratory (results) data and will not include any confidential or sensitive data.

**Table 2:** Clinical strains used to test the multiplex PCR technique.

Study	Population	Cases	Location	Country	Number of *C*. *jejuni*	Reference
Cobra Gold Exercise 1998	Military personnel	Adults	Kanjanaburi, Utapao	Thailand	20	[43]
Cobra Gold Exercise 1999	Military personnel	Adults	Korat	Thailand	83	[43]
Cobra Gold Exercise 2000	Military personnel	Adults	Nakornsrithammarat, Tungsong	Thailand	68	[43]
Cobra Gold Exercise 2001	Military personnel	Adults	Pitsanulok	Thailand	54	[43]
Cobra Gold Exercise 2002	Military personnel	Adults	Sakaew	Thailand	15	[43]
Cobra Gold Exercise 2003	Military personnel	Adults	Pranburi	Thailand	23	[43]
Travelers' diarrhea 2001–02	Travelers, indigenous population	Adults	Bumrungrad International Hosp.	Thailand	51	[43]
Diarrheal surveillance 2004–06	Indigenous Population	Children ≤ 5 Years	Regional Hosp.	Thailand	213	-
Diarrheal surveillance 2008–10	Indigenous Population	Children ≤ 5 Years	Regional Hosp.	Thailand	302	-
Travelers' diarrhea 2001–03	Travelers	Adults	CIWEC Clinic	Nepal	46	-
Diarrheal surveillance 2006–09	Indigenous Population	Children ≤ 5 Years, Adults	Bharatphur, Sukaraj, Kanti, Teku	Nepal	96	-
Diarrheal surveillance 2004–06	Indigenous Population	Children ≤ 5 Years	National Pedriatric Hosp.	Cambodia	25	-

Cary Blair medium were used as transportation medium in these study sites. For *C*. *jejuni* culture and isolation, fresh stool or stool in Carry Blair medium was processed by a modified filtration method [42]. After filtration, the millipore membranes were incubated on BAP at 37°C under microaerobic conditions. The suspected colonies of *C*. *jejuni* were identified by catalase test, nitrate tests and hippurate hydrolysis. Confirmed *C*. *jejuni* isolates were kept frozen at -70°C in 15% glycerol medium.

### Statistical analyses

Statistical analyses presented in this manuscript were calculated using a Chi-square test.

## Results and Discussion

### Description of primers and multiplex mixes

**Fig 3:**
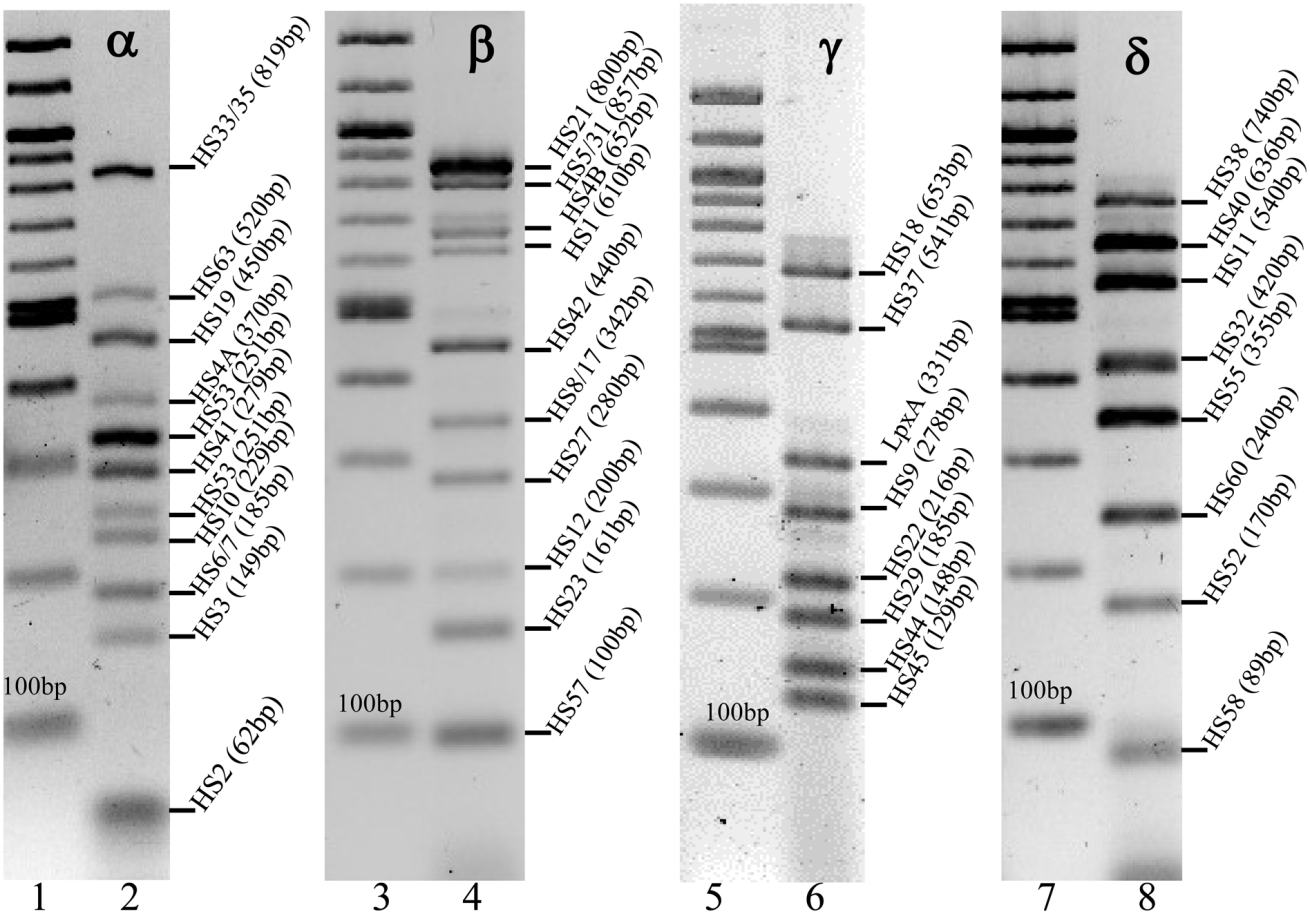
Illustration of PCR amplicons expected when using the updated *C*. *jejuni* CPS multiplex PCR. Lane 1, 100-bp NEB DNA standard; lane 2, mixture of PCR products obtained with all the templates from the alpha mix; lane 3, 100-bp NEB DNA standard; lane 4, mixture of PCR products obtained with all the templates from the beta mix; lane 5, 100-bp NEB DNA standard; lane 6, mixture of PCR products with all the templates from the gamma mix; lane 7, 100-bp NEB DNA standard; lane 8, mixture of PCR products with all the templates from the gamma mix.

The previous *C*. *jejuni* CPS multiplex version was composed of two mixes, alpha and beta, that contained eight and six primer sets, respectively [32]. A total of 23 new primer sets were added for a total of four mixes, alpha, beta, gamma and delta. The alpha mix of the second version contains three additional primers sets (HS19, HS33 and HS63) compared to the initial published version, for a total of 11 primers sets. The beta mix was revised by moving the HS44 primer set to mix gamma and adding five new primer sets (HS5, HS12, HS21, HS27 and HS57). A positive control primer set for *C*. *jejuni* sp. was included in mix gamma following observations that other non-*jejuni Campylobacter* spp. were cross reacting with the multiplex PCR scheme (data not shown). This *C*. *jejuni*-specific primer set amplifies a 331bp region of the *lpxA* gene (involved in lipid A biosynthesis) [44]. Thus, results should be interpreted only if a positive amplification of a 331 bp amplicon is observed in mix gamma. Finally, mix delta contains an additional eight primer mixes. Primers and their respective PCR product sizes are listed in Table 3 and illustrated in Fig 3. All primer sets were tested as described in Materials and Methods.

**Table 3:** Summary of the primer sequences included in the *C*. *jejuni* capsule multiplex typing scheme.

Primers	Product size (bp)	Penner Recognized	Forward sequence	Reverse sequence	Accession number^a^
***Mix Alpha***					
Mu_HS2	62	HS2	CAGCATTGGAGGATTTACAATATAT	CATCCTAGCACAACTCACTTCA	AL111168.1^b^
Mu_HS3	149	HS3	GGTAAGGTTGATTCTGGGTTTAAT	AGATTAGGCCAAGCAATGATAA	HQ343268^c^
Mu_HS4A	370	HS4A^d^	TATATTTGGTTAGGGATCCA	CCTAACATATCATACACTACGGT	HQ343269^c^
Mu_HS6	185	HS6 & HS7	CATACATTTGCTTTCAGATTCTTTAC	ACACGCCTATTGTTGTTGTTC	NC_009839^e^
Mu_HS10	229	HS10	TCTTATGCAGCACGCTGAT	CAAATTCAATCGACTAGCCACT	HQ343271^c^
Mu_HS15	325	HS15, HS31 & HS58	ACAGGTAATAAAATGTGCGAGTTT	ATGCATCTGCAACATCATCC	HQ343272^c^
Mu_HS41	279	HS41	CTTACATATGCTGGTAGAGATGATATG	TGCAATCTCTAAAGCCCAAG	BX545857^f^
Mu_HS53	251	HS53	AGGCAAGCAGGAATTGTTT	TTAATTGCTCTTTGGCAATCTT	CP000025.1^g^
Mu_HS19	450	HS19	CGAGGATGAAAATGCCTCAA	GGCAACAAACAAACATATTCAGA	BX545860^f^
Mu_HS63	522	HS63	AAATTTGTTTTTCATATTTTTACGG	TTAGGTGCGGTTACCAAAGG	KT893438^h^
Mu_HS33	819	HS33 & HS35	GTAGCGGATCAGCAGCATTA	CATCAAAATCATCTTTTAACACCAA	KT893436^h^
***Mix Beta***					
Mu_HS1	610	HS1	TTGGCGGTAAGTTTTTGAAGA	GCAAGAGAAACATCTCGCCTA	BX545859^f^
Mu_HS4B	652	HS4B^i^	GTGGACATGGAACTGGGACT	AAAACGTTTAAAGTCAGTGGAAA	AASY01000000^j^
Mu_HS8	342	HS8 & HS17	TTCACGTGGAGGATTATTGG	TTGAACATTTCATGTGTATTCCCTA	HQ343270^c^
Mu_HS23/36	161	HS23 & HS36	GCTTGGGAGATGAATTTACCTTTA	GCTTTATATCTATCCAGTCCATTATCA	BX545858^f^
Mu_HS42	440	HS42	ATGGTAAAACCGGCATTTCA	ATGCTTCAGTTCCACCCAAA	HQ343274^c^
Mu_HS57	100	HS57	GGGGTAAAATAGCCAATATTCCA	CCAACAAGCCATATTTGTTTTTC	KT893428^h^
Mu_HS12	201	HS12	GGAGGTAAAACGATATTCTCCTTAAA	TGAAGATTTTGAATGGATGTGTG	KT868848^h^
Mu_HS27	280	HS27	GAATAAATATTGCTTCCATACTTTCAA	GCAAAATGAGAATCTCCACCA	KT893437^h^
Mu_HS21	801	HS21	TGGATGGGATATTGATGACAA	CCCTGGAAGAGTATGGGACA	KT868849^h^
Mu_HS31	857	HS5 & HS31	GGCAAAGAGCTTTATTTTGTTGA	GCCGTAGCAACATCAAATACA	KT868847^h^
***Mix Gamma***					
Mu_HS44	148	HS44	AGAAGATGCACTAGGCTCTAG	GCTATCTAATTCCATCCCTG	JF496678^h^
Mu_HS45	128	HS45, HS5, HS32 & HS60	TCCACTTGGGATGAAAAGGA	ACCGCATACTTTGAGCCTGT	KT893432^h^
Mu_HS29	185	HS29	CCCATATTTAAACAATGGAGTGA	TCATACTTTGAAAAACATTATCTGGA	KT868846^h^
Mu_HS22	216	HS22	TCATGGAGCTGGAACAACAG	GCTGGAACTTCTTTTGCAATC	KT893439^h^
Mu_HS9	278	HS9	AAAACTATTAGCTTGATTTTACCTTGG	GCGAAAGACGGATTGTTCAT	KT868844^h^
Mu_HS37	541	HS37	TGGATGAAGGGGACTTATGG	TGGTTTGAAGAGCATCAGCA	KT893431^h^
Mu_HS18	653	HS18	CAGCTATAAATCATGGGTATTGGA	GTAATCAATACATTTTTCCTTGCTT	KT932997^h^
lpxA	331	*C*. *jejuni* sp.	ACAACTTGGTGACGATGTTGTA	CAATCATGDGCDATATGASAATAHGCCAT	[44]
***Mix Delta***					
Mu_HS58	85	HS58 and HS32	TCCGGAAAAATTTTATTTAGATTCTC	AACAATACCAGGATACCAATCTTCA	KT893427^h^
Mu_HS52	170	HS52	AAAACACGCTATTAATCATGGTGAC	ATGTAGGCCAAGTTATACAACCTTTT	KT893429^h^
Mu_HS60	241	HS60	GAAATCATTTTTATGATATTGTGGTT	TCACAGTCACAATAAATAGCCAAA	KT893426^h^
Mu_HS55	341	HS55	GAGATGGTGGTGGTCATCAA	ACGTTGCAACCAATCCTTTG	KT893433^h^
Mu_HS32	420	HS32	GCATACCAGATGGCTTTGG	AATGCAGCGTGCTTCTTATTT	KT893435^h^
Mu_HS11	540	HS11	GAATTGGACATAACCACGGAAT	ATGCAAAGTGCACATATTCTCC	KT868845^h^
Mu_HS40	636	HS40	CAACCCTTGGATGACAATAGAGA	ACCGTCAATATCATCAGGATTTA	KT893434^h^
Mu_HS38	741	HS38	GCCGCAGGAGATAATGAAGA	TTTGCCTTTTAGATCTTGAGGA	KT893430^h^

^*a*^ Genbank accession number of the DNA sequence used to design the primers

^*b*^ Parkhill et al., 2000 [10]

^*c*^ Poly et al., 2011 [32]

^*d*^ HS4A represent HS4, HS13, HS16, HS43, HS50, HS62, HS64 and HS65

^*e*^ Pearson et al., 2007 [19]

^*f*^ Karlyshev et al., 2005 [31]

^*g*^ Fouts et al., 2005 [15]

^*h*^ this study

^*i*^ HS4B includes CG8486, HS16 & HS64

^*j*^ Poly et al., 2007 [14]

### Interpretation of results

As shown in Table 4, some primer sets can show extraneous bands, but the system has been designed to facilitate discrimination. For example, the HS5 template should yield an 857 bp product in mix beta, but it also yields a 129 bp product with the HS45 primer set found in mix gamma. Therefore, if an unknown template yields only a 129 bp product in mix gamma, the CPS type is HS45, but if it yields both a 129 bp product in mix gamma and an 857 bp product in mix beta, the strain is an HS5. Similarly, as described previously [32], HS31 DNA templates should produce a 857 bp product in mix beta, but they also produce a 325 bp product with the HS15 primer set found in mix alpha. The designed HS45 and HS15 primers do not match any sequences of HS5 and HS31 genomes respectively when compared by BLAST algorithm. The reason for these amplifications is still unclear. Nevertheless these additional bands do not interfere with the attribution of HS5 and HS31 CPS types.

**Table 4:** Summary of the *C*. *jejuni* capsule multiplex PCR expected results.

Capsule type	Mix Alpha	Mix Beta	Mix Gamma	Mix Delta
HS1	-	Mu_HS1 (610bp)	-	-
HS2	Mu_HS2 (62bp)	-	-	-
HS3	Mu_HS3 (149bp)	-	-	-
HS4 (HS4 complex)	Mu_HS4A (370bp)	-	-	-
CG8486 (HS4 complex)	-	Mu_HS4B (652bp)	-	-
HS5 (HS5 complex)	-	Mu_HS5 (857bp)	Mu_HS45 (129bp)*	-
HS6 (HS6 complex)	Mu_HS6 (185bp)	-	-	-
HS7 (HS6 complex)	Mu_HS6 (185bp)	-	-	-
HS8 (HS8 complex)	-	Mu_HS8 (342bp)	-	-
HS9	-	-	Mu_HS9 (278bp)	-
HS10	Mu_HS10 (229bp)	-	-	
HS11	-	-	-	Mu_HS11 (540bp)
HS12	-	Mu_HS12 (200bp)	-	-
HS13 (HS4 complex)	Mu_HS4A (370bp)	-	-	-
HS15	Mu_HS15 (325bp)	-	-	-
HS16 (HS4 complex)	Mu_HS4A (370bp)	Mu_HS4B (652bp)	-	Mu_HS52 (170bp)*
HS17 (HS8 complex)	-	Mu_HS8 (342bp)	-	-
HS18	-	-	Mu_HS18 (653bp)	-
HS19	Mu_HS19 (450bp)	-	-	-
HS21	-	Mu_HS21 (800bp)	-	-
HS22	-	-	Mu_HS22 (216bp)	-
HS23 (HS23 complex)	-	Mu_HS23 (161bp)	-	-
HS27	-	Mu_HS27 (280bp)	-	-
HS29	-	-	Mu_HS29 (185bp)	-
HS31 (HS5 complex)	Mu_HS15 (325bp)*	Mu_HS5 (857bp)	-	-
HS32	-	Mu_HS8 (342bp)*	Mu_HS45 (129bp)*	Mu_HS32 (420bp)
HS33 (HS33 complex)	Mu_HS33 (819bp)	-	-	-
HS35 (HS33 complex)	Mu_HS33 (819bp)	-	-	-
HS36 (HS23 complex)	-	Mu_HS23 (161bp)	-	
HS37	-	-	Mu_HS37 (541bp)	
HS38	-	-	-	Mu_HS38 (740bp)
HS40	-	-	-	Mu_HS40 (636bp)
HS41	Mu_HS41 (279bp)	-	-	-
HS42	-	Mu_HS42 (440bp)	-	-
HS43 (HS4 complex)	Mu_HS4A (370bp)	-	-	-
HS44	-	-	Mu_HS44 (148bp)	-
HS45	-	-	Mu_HS45 (129bp)	-
HS50 (HS4 complex)	Mu_HS4A (370bp)	-	-	-
HS52	-	-	-	Mu_HS52 (170bp)
HS53	Mu_HS53 (251bp)	-	-	-
HS55	-	-	-	Mu_HS55 (355bp)
HS57	-	Mu_HS57 (100bp)	-	-
HS58	Mu_HS15 (325bp)*	-	-	Mu_HS58 (89bp)
HS60	-	-	Mu_HS45 (129bp)*	Mu_HS60 (240bp)
HS62 (HS4 complex)	Mu_HS4A (370bp)	-	-	-
HS63	Mu_HS63 (520bp)	-	-	-
HS64 (HS4 complex)	Mu_HS4A (370bp)	Mu_HS4B (652bp)	-	-
HS65 (HS4 complex)	Mu_HS4A (370bp)	-	-	-

(*) un-expected amplification.

During validation, it was also observed that Mu_HS15 primers (325 bp), which were in the alpha mix in the original multiplex, also recognized the HS58 type strain DNA template found in mix delta (89 bp). This observation would suggest that the first generation multiplex was falsely recognizing HS58 strains as HS15. Nucleotide sequence comparisons confirmed a region of homology within the CPS loci of both HS15 and HS58. However, in the current multiplex, amplification of this product does not interfere with attribution of both HS15 or HS58 CPS types, and the Mu_HS15 original primer set was retained in the current multiplex PCR.

Similarly, the Mu_HS8 primers used in the original mix were found to amplify a sugar biosynthesis gene found in the HS32 Penner type strain (Table 4). Thus, the Mu_HS8 primers generate a 342 bp amplicon from mix beta on both strains, but discrimination of HS32 is made by the presence of a 420 bp product (Mu_HS32) in mix delta. In addition, it was observed that HS32 type strain is also recognized by Mu_HS45 primers in mix gamma yielding a 129 bp amplicon. Again because these extraneous amplifications do not interfere with either CPS attribution, the original Mu_HS8 primers were retained in mix beta and Mu_HS32 primers were added to mix delta.

The CPS locus of the type strain of HS45 appeared to be a mosaic of multiple serotypes, an observation that complicated primer design (C. T Parker, unpublished). Primers were designed based on the HS45 sequence with the knowledge that they would also amplify HS5, HS32, and HS60 (Table 4).

### Primers for CPS types in related complexes

Many CPS types fall into related complexes, e. g. HS23 and HS36 (Fig 1). DNA sequencing of the CPS loci had previously revealed that the type strains of HS23 and HS36 share 97.6% DNA sequence identity and >87.9% protein identity [31]. Both strains express the same repeating capsular trisaccharide, but the HS23 type strain was shown to lack the MeOPN modification and one of the four variable heptoses found in HS36 [31]. The primer Mu_HS23 developed in the first multiplex version was retained and does not distinguish HS23 and HS36 capsule types.

The HS4 complex is the largest complex and is composed of eight separate serotypes (HS4, HS13, HS16, HS43, HS50, HS62, HS64 and HS65) (Fig 1) [25]. Only the capsule structure of a clinical isolate from Thailand, CG8486, that typed as HS4/13/64 has been published [30]. The primer set named HS4 (alpha mix, 370bp) and CG8486 (beta Mix, 652bp) in the first publication [32] were re-named HS4A and HS4B, respectively. These primer sets were designed based on the MeOPN transferases present in each strain [14, 32]. Due to the high recombination rate of *C*. *jejuni* strains, isolates that would react with both HS4A and HS4B primers were anticipated and observed (see below). The CPS biosynthesis loci of all 8 type strains in the complex and of CG8486 are highly conserved (C. T Parker, unpublished). Primers HS4A react positively with all eight individual serotypes associated with the HS4 complex, but not with CG8486, as demonstrated in a previous study [32]. Primers HS4B positively recognize CG8486, HS16 and HS64 and this is consistent with the presence of the CG8486 MeOPN transferase-like gene in these three strains (C. T Parker, unpublished). These data suggest that differences among strains within the HS4 complex include differences in the position of MeOPN attachment, an observation that has been confirmed by determination of CPS structure (Monteiro et al, in preparation).

HS5 and HS31 belong to the same complex [25] and are indistinguishable based on their CPS loci gene content (C. T Parker, unpublished). Thus, both HS5 and HS31 are detected by the presence of an 857 bp amplicon in mix beta generated by the Mu_HS5 primers (Table 4).

The CPS loci of the type strains from three additional Penner complexes, HS6/7, HS8/17, and HS33/35, also showed high conservation. The HS8/17 complex was discussed previously [32]. The Mu_HS6 was designed in the previous CPS multiplex PCR version. The HS6 capsule type remains obscure, due to the fact that CPS has been shown not to be the serodeterminant of the HS6 serotype and might explain the high number of false positive identified with Mu_HS6 designed previously [31, 32]. Nevertheless, sequencing of the HS7 Penner type strain showed that the entire HS7 biosynthesis locus was over 99% identical to that of the HS6 type strain (C. T Parker, unpublished). This result corroborates the frequent association of HS6 and HS7 in the Penner typing literature [27]. Finally, it appears that the CPS biosynthesis loci of the type strains of HS33 and HS35 are over 99% identical at the nucleotide level (C. T Parker, unpublished). The Mu_HS33 primer designed in this new version recognizes both serotypes.

The HS1 complex, which includes HS1 and HS44, can also be detected by the same primer set, producing a 610 bp amplicon in mix beta. Additional information on the HS1 complex will be presented separately (F. Poly, unpublished).

Collectively, the sequencing data suggests that strains within these complexes express similar/related CPS structures despite belonging to different serotypes.

### Application of the multiplex to clinical isolates from Thailand

Nine hundred and ninety isolates were positive for the *C*. *jejuni* species-specific gene (*lpxA*) by gamma mix multiplex PCR and six isolates were negative. This result confirms the high level of correlation between the *lpxA* PCR and classic phenotypical methods for the characterization of *C*. *jejuni* sp. [44]. The CPS multiplex PCR assay identified 98% of all 990 *C*. *jejuni* isolates in this study. There were a total of 20 untypeable strains, 17 from Nepal (12 from indigenous population, 5 from travelers), and three from Cambodia, representing 12.8 and 12.5% of the isolates in each respective country (Table 5). This higher of level of non-typeable isolates in those countries does not appear to be random or attributable to method failure. This may indicate presence of localized undefined/unreported capsule types that are not included in the current capsule typing method that was developed largely on strains from North America and Europe [45]. Validation of this hypothesis will require further analysis.

**Table 5:** Capsule multiplex PCR results among all *C*. *jejuni* isolates from different population group from South and South-East Asia. In bold are the five most prevalent CPS types in their respective group. Number in parentheses represent the number of strain in the category.

	Foreign population			Indigenous population			
Capsule type	Travelers Thailand 2001–2002	Military Thailand 1998–2003	Travelers Nepal 2001–2003	Nepal 2006–2009	Cambodia 2004–2006	Thailand 2004–2010	All
HS1/44 complex	2% (1)	4.9% (13)		2.1% (2)	**8.3% (2)**	6.8% (35)	5.4% (53)
HS2	3.9% (2)	**8% (21)**	**17.4% (8)**	**7.4% (7)**	**29.2% (7)**	**19.7% (101)**	**14.7% (146)**
HS3 complex	**13.7% (7)**	3.8% (10)	**10.9% (5)**	**7.4% (7)**	4.2% (1)	**9.2% (47)**	**7.8% (77)**
HS4 complex	5.9% (3)	**22.8% (60)**	**8.7% (4)**	**7.4% (7)**	**12.5% (3)**	**15.8% (81)**	**16.1% (158)**
HS5/31 complex	**15.7% (8)**	5.7% (15)	**8.7% (4)**	**8.5% (8)**	**12.5% (3)**	**13.1% (67)**	**10.6% (105)**
HS6/7 complex			**8.7% (4)**	**11.7% (11)**		1.2% (6)	2.1% (21)
HS8/17 complex	5.9% (3)	5.7% (15)	2.2% (1)	4.3% (4)	**8.3% (2)**	**14.1% (72)**	**9.8% (97)**
HS9	2% (1)		6.5% (3)	2.1% (2)		2.7% (14)	2% (20)
HS10	5.9% (3)	1.5% (4)	2.2% (1)	1.1% (1)	**8.3% (2)**	4.7% (24)	3.5% (35)
HS11	3.9% (2)			2.1% (2)		0.2% (1)	0.5% (5)
HS15	**11.8% (6)**	3.8% (10)	2.2% (1)	1.1% (1)		0.4% (2)	2% (20)
HS21	2% (1)			6.4% (6)		0.2% (1)	0.8% (8)
HS22			4.3% (2)	3.2% (3)			0.5% (5)
HS23/36 complex	**7.8% (4)**	**16.7% (44)**		2.1% (2)		2.3% (12)	6.3% (62)
HS37	5.9% (3)	3.8% (10)	2.2% (1)	1.1% (1)	4.2% (1)	3.1% (16)	3.2% (32)
HS41			6.5% (3)	2.1% (2)			0.5% (5)
HS42		**6.1% (16)**	2.2% (1)	3.2% (3)			2% (20)
HS53	**7.8% (4)**	**14.8% (39)**	2.2% (1)	1.1% (1)		2.7% (14)	6% (59)
HS57				3.2% (3)			0.3% (3)
Other	6% (3)	2.4% (6)	4.4% (2)	9.6% (9)		3.69% (19)	3.9% (39)
Untypeable			10.9% (5)	12.8% (12)	12.5% (3)		2% (20)
Not *C*. *jejuni*				2.1% (2)	4.2% (1)	0.6% (3)	0.6% (6)
Number of strains	51	263	46	96	25	515	996

Overall, as shown in Table 5, the five most common CPS types observed from all sites were the HS4 complex (16.1%), HS2 (14.7%), HS5/31 complex (10.6%), HS8/17 complex (9.8%) and HS3 complex (7.8%). In 2013, Pike and colleagues published a longitudinal study of the most common Penner serotypes worldwide. One of the observations of the study is that HS4 complex, HS2 and HS1/44 complex were the most common serotypes in both developing and developed countries. Surprisingly, our survey demonstrates that HS1/44 type is less frequent in this population, but is still significantly represented with 5.4% of cases in the south and Southeast Asian regions (Table 5).

**Fig 4:**
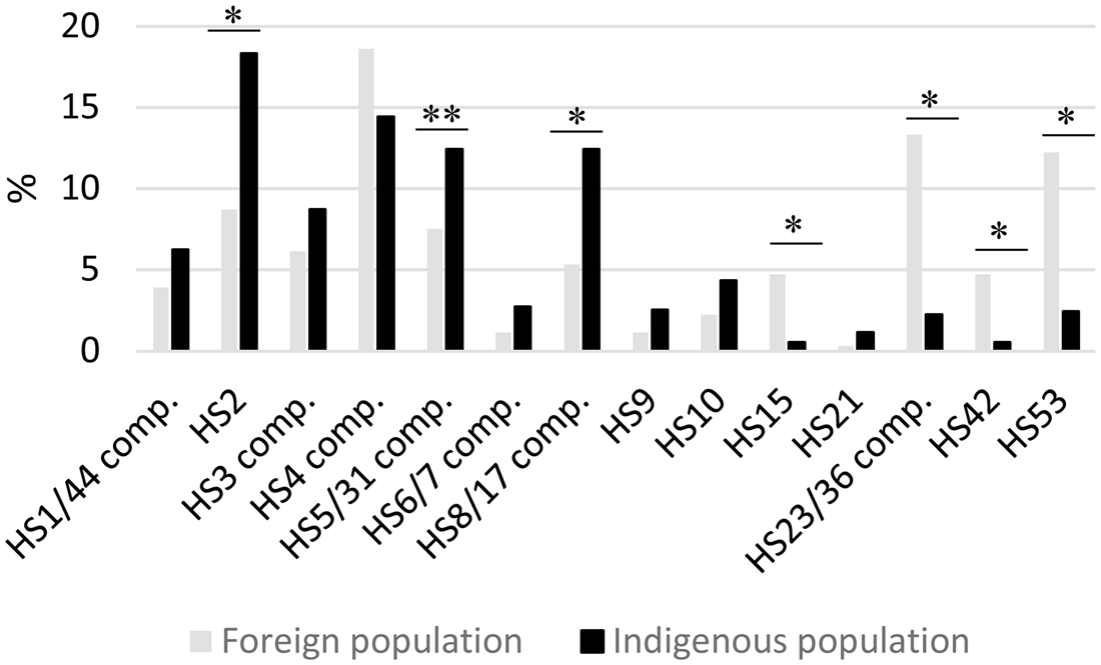
Comparison of the capsule type distribution between indigenous Southeast/South Asia population and foreign population. (*) statistically significant (p<0.01). (**) statistically significant (p<0.05).

Comparison of capsule distribution of total foreign versus indigenous population, shows some noticeable differences (Fig 4). Isolates from CPS types belonging to HS2 (8.7% vs 18.3%, p<0.01), HS5/31 complex (7.5% vs 12.4%, p = 0.019) HS6/7 complex (1.1% vs 2.7%, p = 0.098), HS8/17 complex (5.3 vs 12.4%, p<0.01), HS9 (1.1% vs 2.5%, p = 0.098), are underrepresented in foreign population, while HS15 (4.7% Vs 0.5%, p<0.01), HS23/36 complex (13.3% Vs 2.2%, p<0.01), HS42 (4.7% Vs 0.5%, p<0.01) and HS53 (12.2% Vs 2.4%, p<0.01) are over represented in isolates collected from foreign visitors. A closer look at the foreign population visiting Thailand, travelers and military population, highlight a dichotomy in those two groups (Table 5): The five most common CPS types in non-military travelers to Thailand are the HS5/31 complex (15.7%), HS3 complex (13.7%), HS15 (11.8%) and the HS23/36 (7.8%) and HS53 (7.8%), whereas the five most common CPS types in military personnel deployed to Thailand were the HS4 complex (22.8%), the HS23/36 complex (16.7%), HS53 (14.8%), HS2 (8%), and HS42 (6.1%). The difference of CPS distribution between those two groups is not easy to explain. However, it is likely that isolates from these military exercises had higher clonal relationships than the other isolates studied. The annual military exercises in Thailand take place for one month or less at different locations, and off-duty military personnel may consume the same contaminated foods from local vendors. In addition while there is no differences of the top five most common CPS types between travelers and indigenous population in Nepal, there are noticeable differences between the travelers and indigenous population in Thailand (Table 5). Capsule types HS2, HS4 and HS8/17 complexes are under-represented and HS15, HS53 and HS23/36 complex are over-represented in travelers to Thailand compared to the Thai population (Table 5). While some of those differences can be explained by clonality/outbreaks, the differences are most likely multifactorial and include regional isolation for foreign visitors, differences in food consumed, and/or seasonal changes. Nonetheless, this is important information to take in account for the development of a capsule based vaccine against *C*. *jejuni*.

Finally, no major temporal difference of capsule types distribution was observed in these pediatric populations during 2004–2006 (n = 302) and 2008–2010 (n = 213) time period. *C*. *jejuni* HS2, HS4 complex, HS8/17 complex, HS3, and HS5/31 complex were found as the five most common capsule types, accounting for nearly 70% of isolates in each of these two time periods (data not show).

## Conclusions

The burden of disease caused by *C*. *jejuni* is undeniable. It represents a major health risk for pediatric population living in developing countries as well as travelers visiting those regions. The most promising vaccine approach to alleviate campylobacteriosis in those populations is a capsule conjugate vaccine. A monovalent vaccine targeting HS23/36 CPS type has shown 100% protection in a non-human primate model [33]. To be efficacious a final vaccine should be multivalent and include the most prevalent and most pathogenic *C*. *jejuni* CPS types. In order to gain more information on CPS distribution worldwide we developed a multiplex PCR based approach. In this study we demonstrated the possibility of specifically determining CPS/Penner type through design of specific PCR primer pairs. Typing methods for *C*. *jejuni* remains are limited despite the availability of numerous whole genomes sequences in the last decade. The Penner serotyping method is time and labor intensive, and it is now only performed in a handful of laboratories worldwide. One the major drawbacks in addition to the cost and complexity of the typing sera, is the phase variability of CPS expression [22]. In contrast, the multiplex does not require capsule expression for attribution of capsule type.

A recent systematic review of clinical isolates *C*. *jejuni* Penner typing since the 80’s demonstrate that over 85% of the typed strains were from developed countries. This result demonstrated the lack of information on pediatric population from the developing countries and the necessity to gain more information on CPS type distribution in those region where a CPS conjugate vaccine is the most needed [27]. This method provides a mechanism to address this deficit.

Analysis on almost 1000 clinical isolates collected from 1998 to 2010 in South East Asian peninsula showed that 98% of the *C*. *jejuni* strains were typeable. Knowing the *C*. *jejuni* CPS loci plasticity, the *C*. *jejuni* non-typeable isolates may represent unknown capsules that were not described in the Penner serotype system. Although there are marked differences between the major capsule types reported in the developed world [27] and those reported here from Asia, a limited number of capsule types account for most of the disease. Thus, the 8 most common CPS types account for 76.7% CPS in that region. Thus, these results suggest that the valency required for an effective *C*. *jejuni* conjugate vaccine is similar to that seen in the first pneumococcal conjugate vaccines.

Taken together, these observations validate the development of a multiplex PCR technique. The application of capsule multiplex PCR assay demonstrates simplicity and sensitivity of the technique. All equipment required is standard in most molecular microbiology laboratories. The PCR multiplexing reduces the number of reactions to be performed per samples, and this method is not affected by phase variation of capsule expression. Our study demonstrates the usefulness of the assay in geographical epidemiology for *C*. *jejuni* diarrhea and in describing CPS serotype among clonally related *C*. *jejuni* isolates from spatial distribution and possible outbreak situations.

## References

[1] Preliminary FoodNet data on the incidence of infection with pathogens transmitted commonly through food—10 states, 2006. MMWR MorbMortalWklyRep. 2007;56(14):336–9. 17431379

[2] Taylor DN, Echeverria P, Pitarangsi C, Seriwatana J, Bodhidatta L, Blaser MJ. Influence of strain characteristics and immunity on the epidemiology of Campylobacter infections in Thailand. JClinMicrobiol. 1988;26(5):863–8. 10.1128/jcm.26.5.863-868.1988 PMC266475 3384911

[3] Rao MR, Naficy AB, Savarino SJ, bu-Elyazeed R, Wierzba TF, Peruski LF, et al Pathogenicity and convalescent excretion of Campylobacter in rural Egyptian children. AmJEpidemiol. 2001;154(2):166–73. 10.1093/aje/154.2.166 11447051

[4] Coker AO, Isokpehi RD, Thomas BN, Amisu KO, Obi CL. Human campylobacteriosis in developing countries. EmergInfectDis. 2002;8(3):237–44. 10.3201/eid0803.010233 PMC2732465 11927019

[5] Pimentel M, Chatterjee S, Chang C, Low K, Song Y, Liu C, et al A new rat model links two contemporary theories in irritable bowel syndrome. DigDisSci. 2008;53(4):982–9. 10.1007/s10620-007-9977-z 17934822

[6] Thabane M, Simunovic M, Akhtar-Danesh N, Garg AX, Clark WF, Collins SM, et al An outbreak of acute bacterial gastroenteritis is associated with an increased incidence of irritable bowel syndrome in children. The American journal of gastroenterology. 2010;105(4):933–9. 10.1038/ajg.2010.74 20179687

[7] Pope JE, Krizova A, Garg AX, Thiessen-Philbrook H, Ouimet JM. Campylobacter Reactive Arthritis: A Systematic Review. SeminArthritis Rheum. 2007. 10.1016/j.semarthrit.2006.12.006 PMC2909271 17360026

[8] Ang CW, Jacobs BC, Laman JD. The Guillain-Barre syndrome: a true case of molecular mimicry. Trends Immunol. 2004;25(2):61–6. 15102364 10.1016/j.it.2003.12.004

[9] Lee G, Pan W, Penataro Yori P, Paredes Olortegui M, Tilley D, Gregory M, et al Symptomatic and asymptomatic Campylobacter infections associated with reduced growth in Peruvian children. PLoS Negl Trop Dis. 2013;7(1):e2036 10.1371/journal.pntd.0002036 23383356 PMC3561130

[10] Parkhill J, Wren BW, Mungall K, Ketley JM, Churcher C, Basham D, et al The genome sequence of the food-borne pathogen Campylobacter jejuni reveals hypervariable sequences. Nature. 2000;403(6770):665–8. 10688204 10.1038/35001088

[11] Parker CT, Quinones B, Miller WG, Horn ST, Mandrell RE. Comparative genomic analysis of Campylobacter jejuni strains reveals diversity due to genomic elements similar to those present in C. jejuni strain RM1221. JClinMicrobiol. 2006;44(11):4125–35. 10.1128/JCM.01231-06 PMC1698300 16943349

[12] Dorrell N, Mangan JA, Laing KG, Hinds J, Linton D, Al-Ghusein H, et al Whole genome comparison of Campylobacter jejuni human isolates using a low-cost microarray reveals extensive genetic diversity. Genome Res. 2001;11(10):1706–15. 11591647 10.1101/gr.185801 PMC311159

[13] Poly F, Threadgill D, Stintzi A. Genomic diversity in Campylobacter jejuni: identification of C. jejuni 81-176-specific genes. JClinMicrobiol. 2005;43(5):2330–8. 10.1128/JCM.43.5.2330-2338.2005 PMC1153751 15872262

[14] Poly F, Read T, Tribble DR, Baqar S, Lorenzo M, Guerry P. Genome Sequence of a Clinical Isolate of Campylobacter jejuni from Thailand. InfectImmun. 2007;75(7):3425–33. 10.1128/IAI.00050-07 PMC1932940 17438034

[15] Fouts DE, Mongodin EF, Mandrell RE, Miller WG, Rasko DA, Ravel J, et al Major structural differences and novel potential virulence mechanisms from the genomes of multiple campylobacter species. PLoSBiol. 2005;3(1):e15. 10.1371/journal.pbio.0030015 PMC539331 15660156

[16] Hofreuter D, Tsai J, Watson RO, Novik V, Altman B, Benitez M, et al Unique features of a highly pathogenic Campylobacter jejuni strain. InfectImmun. 2006;74(8):4694–707. 10.1128/IAI.00210-06 PMC1539605 16861657

[17] Poly F, Read TD, Chen YH, Monteiro MA, Serichantalergs O, Pootong P, et al Characterization of two Campylobacter jejuni strains for use in volunteer experimental-infection studies. InfectImmun. 2008;76(12):5655–67. 10.1128/IAI.00780-08 PMC2583572 18809665

[18] Poly F, Threadgill D, Stintzi A. Identification of Campylobacter jejuni ATCC 43431-specific genes by whole microbial genome comparisons. JBacteriol. 2004;186(14):4781–95. 15231810 10.1128/JB.186.14.4781-4795.2004 PMC438563

[19] Pearson BM, Gaskin DJ, Segers RP, Wells JM, Nuijten PJ, van Vliet AH. The complete genome sequence of Campylobacter jejuni strain 81116 (NCTC11828). JBacteriol. 2007;189(22):8402–3. 17873037 10.1128/JB.01404-07 PMC2168669

[20] Grant AJ, Coward C, Jones MA, Woodall CA, Barrow PA, Maskell DJ. Signature-tagged transposon mutagenesis studies demonstrate the dynamic nature of cecal colonization of 2-week-old chickens by Campylobacter jejuni. ApplEnvironMicrobiol. 2005;71(12):8031–41. 10.1128/AEM.71.12.8031-8041.2005 PMC1317333 16332783

[21] Maue AC, Mohawk KL, Giles DK, Poly F, Ewing CP, Jiao Y, et al The polysaccharide capsule of Campylobacter jejuni modulates the host immune response. Infect Immun. 2013;81(3):665–72. 10.1128/IAI.01008-12 23250948 PMC3584872

[22] Bacon DJ, Szymanski CM, Burr DH, Silver RP, Alm RA, Guerry P. A phase-variable capsule is involved in virulence of Campylobacter jejuni 81–176. MolMicrobiol. 2001;40(3):769–77. 10.1046/j.1365-2958.2001.02431.x 11359581

[23] van Alphen LB, Wenzel CQ, Richards MR, Fodor C, Ashmus RA, Stahl M, et al Biological roles of the O-methyl phosphoramidate capsule modification in Campylobacter jejuni. PloS one. 2014;9(1):e87051 10.1371/journal.pone.0087051 24498018 PMC3907429

[24] Keo T, Collins J, Kunwar P, Blaser MJ, Iovine NM. Campylobacter capsule and lipooligosaccharide confer resistance to serum and cationic antimicrobials. Virulence. 2011;2(1):30–40. 21266840 10.4161/viru.2.1.14752 PMC3073237

[25] Preston MA, Penner JL. Characterization of cross-reacting serotypes of Campylobacter jejuni. CanJMicrobiol. 1989;35(2):265–73. 10.1139/m89-040 2472859

[26] Karlyshev AV, Linton D, Gregson NA, Lastovica AJ, Wren BW. Genetic and biochemical evidence of a Campylobacter jejuni capsular polysaccharide that accounts for Penner serotype specificity. MolMicrobiol. 2000;35(3):529–41. 10.1046/j.1365-2958.2000.01717.x 10672176

[27] Pike BL, Guerry P, Poly F. Global Distribution of Penner Serotypes: A Systematic Review. PloS one. 2013;8(6):e67375. 23826280 10.1371/journal.pone.0067375 PMC3694973

[28] Maue AC, Poly F, Guerry P. A capsule conjugate vaccine approach to prevent diarrheal disease caused by Campylobacter jejuni. Human vaccines & immunotherapeutics. 2014;10(6):1499–504. 24632556 10.4161/hv.27985 PMC5396224

[29] Guerry P, Poly F, Riddle M, Maue AC, Chen YH, Monteiro MA. Campylobacter polysaccharide capsules: virulence and vaccines. Front Cell Infect Microbiol. 2012;2:7 10.3389/fcimb.2012.00007 22919599 PMC3417588

[30] Chen YH, Poly F, Pakulski Z, Guerry P, Monteiro MA. The chemical structure and genetic locus of Campylobacter jejuni CG8486 (serotype HS:4) capsular polysaccharide: the identification of 6-deoxy-D-ido-heptopyranose. CarbohydrRes. 2008;343(6):1034–40. 10.1016/j.carres.2008.02.024 18346720

[31] Karlyshev AV, Champion OL, Churcher C, Brisson JR, Jarrell HC, Gilbert M, et al Analysis of Campylobacter jejuni capsular loci reveals multiple mechanisms for the generation of structural diversity and the ability to form complex heptoses. MolMicrobiol. 2005;55(1):90–103. 10.1111/j.1365-2958.2004.04374.x 15612919

[32] Poly F, Serichatalergs O, Schulman M, Ju J, Cates CN, Kanipes M, et al Discrimination of major capsular types of Campylobacter jejuni by multiplex PCR. JClinMicrobiol. 2011;49(5):1750–7. 10.1128/JCM.02348-10 PMC3122684 21411576

[33] Monteiro MA, Baqar S, Hall ER, Chen YH, Porter CK, Bentzel DE, et al Capsule polysaccharide conjugate vaccine against diarrheal disease caused by Campylobacter jejuni. InfectImmun. 2009;77(3):1128–36. 10.1128/IAI.01056-08 PMC2643618 19114545

[34] Bertolo L, Ewing CP, Maue A, Poly F, Guerry P, Monteiro MA. The design of a capsule polysaccharide conjugate vaccine against Campylobacter jejuni serotype HS15. Carbohydr Res. 2013;366:45–9. 10.1016/j.carres.2012.11.017 23261782

[35] Lesinski GB, Westerink MA. Vaccines against polysaccharide antigens. Current drug targets Infectious disorders. 2001;1(3):325–34. 12455405 10.2174/1568005014605964

[36] Penner JL, Hennessy JN, Congi RV. Serotyping of Campylobacter jejuni and Campylobacter coli on the basis of thermostable antigens. EurJClinMicrobiol. 1983;2(4):378–83. 10.1007/BF02019474 6628376

[37] Palmer SR, Gully PR, White JM, Pearson AD, Suckling WG, Jones DM, et al Water-borne outbreak of campylobacter gastroenteritis. Lancet. 1983;1(8319):287–90. 6130305 10.1016/s0140-6736(83)91698-7

[38] Parker CT, Horn ST, Gilbert M, Miller WG, Woodward DL, Mandrell RE. Comparison of Campylobacter jejuni lipooligosaccharide biosynthesis loci from a variety of sources. JClinMicrobiol. 2005;43(6):2771–81. 10.1128/JCM.43.6.2771-2781.2005 PMC1151924 15956396

[39] Korlath JA, Osterholm MT, Judy LA, Forfang JC, Robinson RA. A point-source outbreak of campylobacteriosis associated with consumption of raw milk. JInfectDis. 1985;152(3):592–6. 10.1093/infdis/152.3.592 4031557

[40] Miller WG, Bates AH, Horn ST, Brandl MT, Wachtel MR, Mandrell RE. Detection on surfaces and in Caco-2 cells of Campylobacter jejuni cells transformed with new gfp, yfp, and cfp marker plasmids. ApplEnvironMicrobiol. 2000;66(12):5426–36. 10.1128/aem.66.12.5426-5436.2000 PMC92478 11097924

[41] Untergasser A, Cutcutache I, Koressaar T, Ye J, Faircloth BC, Remm M, et al Primer3—new capabilities and interfaces. Nucleic acids research. 2012;40(15):e115 22730293 10.1093/nar/gks596 PMC3424584

[42] Steele TW, McDermott SN. The use of membrane filters applied directly to the surface of agar plates for the isolation of Campylobacter jejuni from feces. Pathology. 1984;16(3):263–5. 6393011 10.3109/00313028409068535

[43] Serichantalergs O, Pootong P, Dalsgaard A, Bodhidatta L, Guerry P, Tribble DR, et al PFGE, Lior serotype, and antimicrobial resistance patterns among Campylobacter jejuni isolated from travelers and US military personnel with acute diarrhea in Thailand, 1998–2003. Gut pathogens. 2010;2(1):15 10.1186/1757-4749-2-15 21062505 PMC2989297

[44] Klena JD, Parker CT, Knibb K, Ibbitt JC, Devane PM, Horn ST, et al Differentiation of Campylobacter coli, Campylobacter jejuni, Campylobacter lari, and Campylobacter upsaliensis by a multiplex PCR developed from the nucleotide sequence of the lipid A gene lpxA. JClinMicrobiol. 2004;42(12):5549–57. 10.1128/JCM.42.12.5549-5557.2004 PMC535264 15583280

[45] Penner JL, Hennessy JN. Passive hemagglutination technique for serotyping Campylobacter fetus subsp. jejuni on the basis of soluble heat-stable antigens. JClinMicrobiol. 1980;12(6):732–7. 10.1128/jcm.12.6.732-737.1980 PMC273687 6796598

