# Correction: Updated *Campylobacter jejuni* Capsule PCR Multiplex Typing System and Its Application to Clinical Isolates from South and Southeast Asia

**DOI:** 10.1371/journal.pone.0151410

**Published:** 2016-03-07

**Authors:** Frédéric Poly, Oralak Serichantalergs, Janelle Kuroiwa, Piyarat Pootong, Carl Mason, Patricia Guerry, Craig T. Parker

The authors wish to correct the Competing Interests statement to state that authors Frédéric Poly, Patricia Guerry, and Craig T. Parker hold a patent related to the multiplex PCR technique presented in this article, "Multiplex amplification reaction method for determination of *Campylobacter jejuni* Penner/capsule type" (US 20150044682 A1). Because the patent rights belong to the United States Navy, the authors were unaware that this patent should be disclosed.
